# Moving to Universal Coverage? Trends in the Burden of Out-Of-Pocket Payments for Health Care across Social Groups in India, 1999–2000 to 2011–12

**DOI:** 10.1371/journal.pone.0105162

**Published:** 2014-08-15

**Authors:** Anup Karan, Sakthivel Selvaraj, Ajay Mahal

**Affiliations:** 1 Indian Institute of Public Health Gandhinagar, Gujarat, India; 2 Nuffield Department of Population Health, University of Oxford, Oxford, United Kingdom; 3 Public Health Foundation of India, New Delhi, India; 4 School of Public Health and Preventive Medicine, Monash University, Melbourne, Australia; University of Washington, United States of America

## Abstract

In the background of ongoing health sector reforms in India, the paper investigates the magnitude and trends in out-of-pocket and catastrophic payments for key population sub-groups. Data from three rounds of nationally representative consumer expenditure surveys (1999–2000, 2004–05 and 2011–12) were pooled to assess changes over time in a range of out-of-pocket -related outcome indicators for the poorest 20% households, scheduled caste and tribe households and Muslims households relative to their better-off/majority religion counterparts. Our results suggest that the poorest 20% of households experienced a decline in the proportion reporting any OOP for inpatient care relative to the top 20% and Muslim households saw an increase in the proportion reporting any inpatient OOP relative to non-Muslim households during 2000-2012. The change in the proportion of Muslim households or SC/ST households reporting any OOP for outpatient care was similar to that for their respective more advantaged counterparts; but the poorest 20% of households experienced a faster increase in the proportion reporting any OOP for outpatient care than their top 20% counterparts. SC/ST, Muslim and the poorest 20% of households experienced as faster increase in the share of outpatient OOP in total household spending relative to their advantaged counterparts. We conclude that the financial burden of out of pocket spending increased faster among the disadvantaged groups relative to their more advantaged counterparts. Although the poorest 20% saw a relative decline in OOP spending on inpatient care as a share of household spending, this is likely the result of foregoing inpatient care, than of accessing benefits from the recent expansion of cashless publicly financed insurance schemes for inpatient care. Our results highlight the need to explore the reasons underlying the lack of effectiveness of existing public health financing programs and public sector health services in reaching less-advantaged castes and religious minorities.

## Introduction

The World Health Report 2000 identified financial protection against the costs of ill health as a fundamental objective of health systems, on the premise that a fair health system ensures households make health care payments according to their ability to pay rather than the risk of illness [1]. Financial protection from direct payments in order to access health care is also a key element of ‘universal coverage’ [2]. This goal is especially salient in developing countries whose populations tend to rely heavily on out-of-pocket (OOP) payments to finance their healthcare [1], [3].

Equity in access to care and financing is a key policy concern in India, as suggested in multiple policy documents, including most recently, a policy report of an expert group on universal health coverage [4]–[6]. This is unsurprising, given that the Indian health care system is characterized by a significant private sector accounting for nearly 80% of all outpatient visits and more than half of all hospital stays [7], [8]. The private sector provides mainly curative care on a fee for service basis (as used here, the “private sector” consists of ‘for-profit’ hospitals, ‘not-for-profit’ (non-government organisations [NGO], charitable institutions, trusts, etc.) institutions and private individual practitioners [7]. In the public sector, central and state governments provide publicly financed and managed curative, preventive and promotive health services, including high-end tertiary care, at highly subsidized rates. Multiple previous studies have suggested that the heavy dependence of the Indian health system on OOP payments as mean of financing health care has contributed significantly to household impoverishment in the country [9]–[16].

Governments at the centre and in a few states in India have introduced both supply- and demand-effort interventions to improve the affordability of and physical access to health care. The ‘National Rural Health Mission’ (NRHM) was initiated in 2005 to improve the availability of healthcare services in rural India, primarily by improved funding for existing public sector facilities [17]. Demand side interventions include provision of direct financial assistance and new risk pooling mechanisms. A publicly funded health insurance scheme for the poor supported by central and state funds, the ‘*Rashtriya Swasthya Bima Yojana*’ (RSBY), was initiated in 2008 and is currently amongst the largest such schemes in the world [18]. There are state level publicly funded insurance initiatives as well, including the ‘*Yeshasvini*’ and ‘*Vajpayee Aarogyasri*’ health insurance schemes (Karnataka), the ‘*Rajiv Aarogyasri*’ scheme (Andhra Pradesh) and the Chief Minister's Insurance Scheme for Life Saving Treatment (Tamil Nadu), most intended to help groups with low socioeconomic status [5]–[6], [19].

Few recent studies have focused on the equity dimensions of OOP health spending in India and most existing analyses rely on an examination of single-period cross-sectional data. One set of analyses highlight that richer households pay more OOP per capita for healthcare, but as a proportion of ‘ability to pay’, OOP expenses tend to be greater for poorer households [13], [20]. A second strand of the literature has focused on caste and rural-urban differentials in OOP expenses incurred by households. Bonu et al. [11] using nationally representative survey data of the year 2004–05, found that agricultural households, and households from scheduled castes (SC) and other backward classes (OBCs) incurred higher catastrophic OOP health payments than households belonging to other castes and urban-based households. A study of 550 households in Kerala concluded that households belonging to less-advantaged castes experience higher shares of OOP in total income, although noting that for chronic conditions, less-advantaged castes spent less OOP relative to their advantaged caste counterparts [21]. However, results from these analyses are unable to shed much light on trends in the burden of OOP on the poor and other disadvantaged groups, particularly over the last decade. In fact, we are aware of only one study which attempted to do this for the period 2005–2010 for groups ranked by economic status [22], concluding that the poorest 20% of Indian households witnessed faster increases in the share of households incurring catastrophic OOP payments than richer households, even in districts where the government subsidized public insurance programs had been rolled out. However, many of the government programs, particularly publicly funded insurance, were still in an incipient stage at the time of this earlier work.

We assess changes in the OOP burden on the poor and other socially less advantaged populations relative to their better-off counterparts in India over the period from 2000 to 2012 after pooling 3 waves of household-level consumption expenditure data from the National Sample Survey Organisation (NSSO). This paper contributes to the literature on financial burden of health care in three ways. First, it presents trends in multiple indicators of the burden of OOP spending by poor and disadvantaged groups over a longer period, from 2000 to 2012, compared to earlier work. Thus, the paper can help provide a more careful assessment of the on-going demand- and supply-side public sector investments in terms of their equity implications. Second, our analysis covers a larger group of social groups than analysed in previous work. In addition to groups ranked by economic status and caste, we also consider religious minorities, particularly Muslims, who have been the subject of a recent major policy report [23]. We are also able to test for inter-group differences in trends in OOP indicators and better control for confounders using multivariate analysis.

## Methods

### Data

The data for this study are drawn from nationally representative consumer expenditure surveys (CES), conducted by the National Sample Survey Organisation (NSSO) in 3 quinquennial (five yearly) rounds: 1999–2000, 2004–05 and 2011–12. The number of households surveyed during the three surveys varied between 100 and 124 thousand, with approximately 70 per cent of sample households located in rural areas. This paper is based on anonymized survey data collected by the National Sample Survey organization (NSSO), a department of the Indian Ministry of Statistics and Programme Implementation. The data is available to Indian and international public at a pre announced price. We have bought and obtained permission from the NSSO to use this data in our research.

The CES gathers information on households' consumption expenditure for roughly 350 food and non-food items. Under the non-food category, the survey collects household OOP expenditure for inpatient and outpatient medical spending using different recall periods. These are covered under item numbers 410 to 419 and 420 to 429 for institutional and non-institutional health expenditure respectively in the survey questionnaire. This study uses data on OOP health spending on outpatient care using a 30-day recall period and on inpatient care using a 365-day recall period in the three surveys.

In addition to household expenditure, the survey data include information on a range of socio-economic and demographic characteristics of households. These include caste (Scheduled Caste, Scheduled Tribe, Other Backward Classes, and Others), religion (Hindu, Muslims, Christian and 5 other religions), main source of livelihood for household members (whether self-employed, agriculture labour, holding a regular job, etc.), place of residence (state and whether rural or urban), household size, gender, age, marital status and educational attainment of individual household members.

### Outcome Variables

We used multiple indicators of the financial burden of ill-health using information on OOP expenditure on healthcare incurred by households. The specific indicators used were: (a) monthly OOP spending (at constant 1999–2000 prices) per household member, (b) share of OOP in total household spending, and (c) the proportion of households incurring catastrophic levels of OOP spending by: i) OOP being more than 10% of total household expenditure; and ii) OOP being more than 25% of the total non-food expenditure. The specific thresholds used by us reflect two features of the literature: the use of two types of denominators for constructing measures of catastrophic spending: non-food spending and total spending; and a range of ratios of health spending (to the specific denominator) as thresholds to construct measures of catastrophic spending. In our paper, we use thresholds that lie roughly in the mid-point for each type in the literature. As a robustness check, we also undertook additional analyses by varying thresholds for OOP as a share of total expenditure and non-food expenditure. Analyses were separately conducted for OOP spending on inpatient care, outpatient care and all types of care taken together (Total OOP).

In addition, we used indicators for whether a household incurred any OOP spending, separately for inpatient care, outpatient care and all types of care. Although the survey data we used did not include any information on healthcare use, these indicators served as proxies for utilization of healthcare services. This interpretation is appropriate given that 70%–75% of all health-care spending in India is financed via OOP by households. Indeed, one recent survey (healthcare utilization and expenditure survey of the National sample Survey Organization implemented in 2004) that contained information both on healthcare use and OOP showed that the share of households with a member reporting ‘outpatient care use' was very close to the share of households reporting ‘any OOP on outpatient care’ (27.5% versus 26.0%), with a correlation coefficient between the two indicators of 0.95. Similarly, 10.9 per cent of households reported a member being hospitalized in the last one year and 10.8 per cent of the households reported incurring “any OOP on inpatient care”, with a correlation coefficient between the two indicators of 0.99. Our approach of using a report of any OOP spending as a proxy for utilization follows seminal studies such as Manning et al. [24] that used data from the Rand Health Insurance Experiment in the United States. Subsequent studies have also relied on a similar interpretation of the indicator variable for incurring any OOP spending [25], [26], [27].

We assessed trends in the burden of OOP spending across three dimensions of household socio-economic status (SES): economic rank based on household spending per capita, caste status and religion. Our measure of economic position was a household's quintile ranking based on expenditure per capita (after using sample weights), separately for rural and urban populations. Caste is an informal (but pervasive) system social hierarchy in Indian society. Less-advantaged caste populations are generally acknowledged to have undergone various forms of social discrimination over centuries. India provides various forms of affirmative action to protect less-advantaged caste groups. For this purpose, these caste groups have been classified into Scheduled Tribes (ST), Scheduled Castes (SC) and other backward classes (OBC), in ascending order of hierarchy, under the Indian constitution. The caste affiliation of households was determined by their response to a question inquiring whether they belonged to Scheduled Tribes (STs), Scheduled Castes (SCs) other backward classes (OBCs) and “Other” category. Among the religion categories, we compared Muslim households with non-Muslim (primarily Hindu) households. Other religions include Christian, Sikh, Buddhism, Jainism, Zoroastrian and other small religious communities.

### Regression specification

Our primary goal was to compare the performance of less advantaged households with their more advantaged counterparts with respect to multiple indicators of the financial burden described earlier. Two equations were estimated using a pooled dataset comprising data from all three CES survey rounds. The first (simple) specification is described in (1):
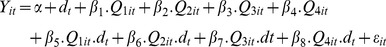
(1)


Here Y_it_ is the outcome indicator for household ‘i’ at time ‘t’ (an indicator of OOP spending), d_t_ stands for the time dummy (‘t1’ for the year 2004–05 and ‘t2’ for the year 2011–12 as against ‘t0’ for the year 1999–2000), ‘*Q1*’ to ‘*Q4*’ represent time specific dummies for each of quintile groups, with the richest quintile being the excluded group. The coefficients of the interaction terms – ‘Q_it_.d_t_’ denote changes in outcomes over time for the various (less well off) quintile groups relative to the richest group. The actual magnitude of the outcome for the richest quintile in the baseline period of 1999–2000 is represented by the constant term ‘*α*’ in equation (1) and ‘*ε*_it_’ is the usual error term.

A version of equation (1) was estimated for the other two indicators of socioeconomic status we used, i.e., the caste and religion affiliation of households. Equation (1) considered only one set of socioeconomic rankings and the estimated trends are likely to be confounded by other indicators of social ranking and other socio-economic and demographic correlates of healthcare use and spending. For example, the share of elderly members and young children in the household and place of residence (rural or urban) are likely to be strongly positively correlated with healthcare use and spending by households. Thus we considered a second expanded specification that included multiple indicators of household social ranking and socioeconomic characteristics. In addition, to capture any unobserved state-specific characteristics that might influence outcomes, we also included state fixed effects. The final equation, including all three different dimensions of socio-economic status of interest, the interaction terms of socio-economic status with time dummies, other correlates and state level fixed effects is as follows:
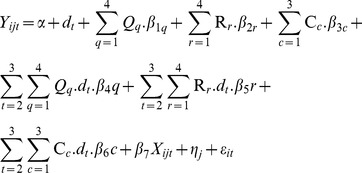
(2)


In equation (2), the additional terms ‘R_r_’ and ‘C_c_’ represent religion and caste groups of households. Accordingly, the new additional interaction terms R_r_.dt, and C_c_.dt represent the estimates for the relative changes in the outcome indicators for the religion and caste groups for 2005 (t1) and 2012 (t2), starting from the baseline of 2000 (t0). X_ijt_ represents a set of other socio-demographic covariates likely to be associated with the changes on OOP related outcome indicators. The additional subscript ‘j’ stands for state level variations and ‘η_j_‘ denotes state level fixed effects. The ’other’ covariates in the analysis include household size, main source of livelihood, of household, religion, the ratio of males to females in the household, the proportion of children in the household, the proportion of elderly in the household, proportion of married members in the household, and rural/urban location. Summary statistics are presented in Table S1.

Both equation (1) and equation (2) are estimated as ‘linear probability models’ when the outcome indicators are indicator variables, as when the dependent variables are whether a household reported “any OOP”, “any inpatient (outpatient) OOP” or any catastrophic OOP spending. Estimates based on equation (2) were used to assess relative changes in outcomes for the most disadvantaged household groups (poorest quintile, being Muslim and schedule caste/tribe (SC/ST) as against changes for their respective best placed counterparts.

## Results

Table 1 and Table 2 report descriptive statistics on key outcomes by place of residence (rural or urban) and socio-economic groups. The data in Table 1 show that the share of households reporting any OOP spending first declined (in 2005) in comparison to 2000, both in rural and urban areas (by about 4 to 5 percentage points) but then increased in 2012, relative to their 2005 share, by more than 11 percentage points in rural areas and 9 percentage points in urban areas. The decline in the share of households reporting any OOP spending between 2000 and 2005 was due mainly to declines in the reporting of any inpatient expenditure, with the share of households reporting any outpatient OOP spending remaining roughly unchanged between 2000 and 2005. However, the rise in the share of households reporting any OOP spending post-2005 was accompanied by increased household shares reporting OOP spending for both outpatient and inpatient care.

**Table 1:** Mean outcome indicators in rural and urban India in the 2000, 2005 and 2012.

	Rural			Urban		
	2000	2005	2012	2000	2005	2012
**Percentage of households reporting OOP**						
Inpatient	18.48 [18.19, 18.76]	8.95 [8.75, 9.15]	14.02 [13.74, 14.30]	19.35 [19.01, 19.70]	9.76 [9.48, 10.03]	14.76 [14.42, 15.10]
Outpatient	61.85 [61.50, 62.21]	61.44 [61.10, 61.79]	78.76 [78.43, 79.09]	61.36 [60.93, 61.79]	62.86 [62.42, 63.31]	75.92 [75.52, 76.33]
Total OOP	69.82 [69.48, 70.15]	64.04 [63.71, 64.38]	81.36 [81.05, 81.68]	69.10 [68.69, 69.51]	65.41 [64.97, 65.84]	78.52 [78.13, 78.92]
**Per person monthly OOP (INR) at constant 1999–2000 prices** *						
Inpatient	6.66 [6.38, 6.94]	8.50 [8.02, 8.98]	15.07 [14.18, 15.95]	12.33 [11.45, 13.20]	13.24 [12.16, 14.32]	25.03 [23.64, 26.42]
Outpatient	22.93 [22.35, 23.50]	24.18 [23.73, 24.63]	31.98 [31.33, 32.64]	30.95 [29.99, 31.91]	34.12 [33.31, 34.92]	47.33 [46.27, 48.39]
Total OOP	29.59 [28.92, 30.25]	32.68 [31.98, 33.38]	47.05 [45.87, 48.23]	43.28 [41.91, 44.65]	47.36 [45.95, 48.77	72.36 [70.48, 74.24]
**OOP as share (%) of household expenditure**						
Inpatient	1.30 [1.26, 1.34]	1.59 [1.54, 1.63]	2.36 [2.29, 2.42]	1.31 [1.26, 1.35]	1.32 [1.26, 1.38]	1.86 [1.79, 1.92]
Outpatient	4.74 [4.68, 4.80]	4.75 [4.70, 4.81]	5.37 [5.31, 5.44]	3.60 [3.54, 3.66]	3.73 [3.67, 3.79]	3.89 [3.83, 3.95]
Total OOP	6.05 [5.97, 6.12]	6.34 [6.26, 6.41]	7.73 [7.63, 7.82]	4.91 [4.82, 4.99]	5.05 [4.96, 5.14]	5.74 [5.65, 5.84]
**Percentage of household reporting OOP share>10% of household expenditure**						
Inpatient	2.48 [2.36, 2.59]	3.18 [3.06, 3.31]	4.85 [4.68, 5.02]	2.71 [2.57, 2.85]	3.14 [2.98, 3.30]	4.66 [4.46, 4.86]
Outpatient	11.44 [11.21, 11.68]	11.60 [11.38, 11.82]	13.31 [13.03, 13.58]	8.50 [8.25, 8.75]	9.53 [9.25, 9.80]	9.91 [9.63, 10.20]
Total OOP	15.00 [14.74, 15.27]	15.07 [14.82, 15.31]	18.86 [18.55, 19.18]	12.36 [12.07, 12.65]	13.26 [12.95, 13.58]	15.86 [15.51, 16.20]
**Percentage of household reporting OOP share>25% of non-food expenditure**						
Inpatient	2.01 [1.91, 2.11]	3.13 [3.01, 3.25]	4.28 [4.11, 4.44]	1.89 [1.77, 2.01]	2.70 [2.55, 2.84]	3.63 [3.45, 3.81]
Outpatient	10.99 [10.76, 11.22]	8.91 [8.71, 9.11]	8.29 [8.07, 8.51]	5.95 [7.74, 6.16]	5.47 [5.26, 5.68]	4.64 [4.44, 4.84]
Total OOP	14.70 [14.44, 14.96]	15.21 [14.96, 15.46]	15.36 [15.07, 15.65]	9.29 [9.04, 9.55]	10.59 [10.31, 10.88]	10.84 [10.54, 11.13]

Source: Authors’ estimates from respective NSSO surveys.

Notes: Figures in brackets represent 95% confidence interval;

*State level average consumer price indices (CPI) separately for rural (CPI for rural labour) and urban areas (CPI for industrial workers) were used to convert the OOP values at constant 1999–2000 prices.

**Table 2:** Percentage of households reporting OOP, share (%) of OOP in total household expenditure and percentage of households reporting catastrophic expenditure among low and high SES households separately for total OOP, inpatient and outpatient expenditure, 2000 and 2012.

	Poorest 20%		Richest 20%		SC/ST		Other caste		Muslim		Hindu	
Outcome	2000	2012	2000	2012	2000	2012	2000	2012	2000	2012	2000	2012
**Percentage of households reporting**												
Inpatient	14.6 [14.1, 15.1]	9.4 [8.9, 9.8]	24.6 [24.1, 25.1]	22.0 [21.5, 22.5]	18.2 [17.8, 18.6]	12.6 [12.3, 13.0]	20.6 [20.3, 21.0]	15.7 [15.3, 16.1]	18.6 [17.96, 19.22]	14.7 [14.05, 15.26]	18.6 [18.34, 18.84]	14.1 [13.82, 14.31]
Outpatient	51.3 [50.7, 52.0]	73.3 [72.7, 73.9]	68.2 [67.7, 68.8]	79.6 [79.1, 80.1]	58.0 [57.4, 58.5]	75.1 [74.7, 75.6]	64.2 [63.8, 64.7]	80.3 [79.9, 80.7]	66.49 [65.7, 67.25]	82.76 [82.1, 83.40]	60.9 [60.57, 61.2]	77.22 [76.9, 77.52]
Total OOP	60.0 [59.3, 60.7]	75.9 [75.3, 76.5]	75.9 [75.4, 76.4]	83.0 [82.5, 83.5]	66.7 [66.17, 67.2]	78.1 [77.6, 78.6]	72.2 [71.8, 72.6]	82.7 [82.3, 83.1]	73.8 [73.11, 74.54]	84.7 [84.12, 85.35]	68. 9 [68.6, 69.19]	79.9[79.59, 80.16]
**Per person monthly OOP (INR) at constant 1999–2000 prices**												
Inpatient	1.4 [1.3, 1.5]	9.4 [8.9, 9.8]	24.6 [24.1, 25.1]	22.0 [21.5, 22.5]	4.93 [4.60, 5,26]	10.72 [9.87, 11.56]	11.2 [10.4, 11.9]	27.3 [25.4, 29.2]	6.53 [5.65, 7.42]	14.58 [13.0, 16.13]	7.96 [7.59, 8.3]	17.8 [16.98, 18.7]
Outpatient	8.2 [8.0, 8.4]	73.3 [72.7, 73.9]	68.2 [67.7, 68.8]	79.6 [79.1, 80.1]	18.98 [18.4, 19.5]	27.5 [26.9, 28.2]	30.1 [29.1, 31.1]	45.6 [44.4, 46.8]	23.9 [22.75, 25.13]	33.76 [31.7, 35.80]	24.78 [24.2, 25.36]	35.9 [35.27, 36.46]
Total OOP	9.5 [9.3, 9.7]	75.9 [75.3, 76.5]	75.9 [75.4, 76.4]	83.0 [82.5, 83.5]	23.9 [23.3, 24.6]	38.3 [37.1, 39.4]	41.3 [40.0, 42.5]	72.9 [70.5, 75.3]	30.48 [28.9, 32.03]	48.35 [45.5, 51.17]	32.75 [32.0, 33.47]	53.7 [52.60, 54.79]
**OOP as share (%) of household expenditure**												
Inpatient	0.45 [0.42, 0.47]	0.64 [0.60, 0.68]	1.99 [1.92, 2.07]	3.36 [3.23, 3.49]	1.06 [1.02, 1.11]	1.83 [1.75, 1.91]	1.39 [1.34, 1.44]	2.28 [2.20, 2.37]	1.13 [1.06, 1.21]	1.92 [1.81, 2.04]	1.29 [1.26, 1.33]	2.10 [2.05, 2.15]
Outpatient	2.76 [2.70, 2.82]	3.48 [3.42, 3.55]	5.19 [5.07, 5.31]	5.16 [5.05, 5.27]	4.16 [4.08, 4.24]	4.75 [4.67, 4.83]	4.04 [3.98, 4.11]	4.30 [4.23, 4.38]	4.35 [4.23, 4.48]	5.10 [4.95, 5.24]	4.31 [4.25, 4.36]	4.59 [4.53, 4.64]
Total OOP	3.21 [3.14, 3.28]	4.12 [4.05, 4.20]	7.18 [7.04, 7.33]	8.52 [8.34, 8.69]	5.23 [5.13, 5.32]	6.57 [6.46, 6.69]	5.43 [5.35, 5.52]	6.59 [6.47, 6.71]	5.49 [5.33, 5.64]	7.02 [6.83, 7.21]	5.60 [5.54, 5.66]	6.68 [6.61, 6.76]
**Catastrophic expenditure at 10% of household expenditure**												
Inpatient	0.76 [0.64, 0.88]	1.77 [1.58, 1.96]	5.59 [5.33, 5.85]	10.17 [9.80, 10.55]	1.91 [1.76, 2.06]	3.82 [3.60, 4.04]	2.75 [2.61, 2.90]	5.53 [5.28, 5.77]	2.09 [1.86, 2.32]	4.25 [3.90, 4.59]	2.53 [2.43, 2.63]	4.84 [4.69, 4.99]
Outpatient	6.80 [6.45, 7.14]	8.04 [7.65, 8.42]	15.3 [14.9, 15.7]	16.7 [16.22, 17.2]	10.16 [9.83, 10.49]	11.8 [11.44, 12.18]	10.06 [9.80, 10.33]	11.3 [10.93, 11.62]	10.7 [10.22, 11.22	12.3 [11.71, 12.83]	10.7 [10.48, 10.88]	12.2 [11.94, 12.40]
Total OOP	8.19 [7.8, 8.6]	10.8 [10.4, 11.3]	21.9 [21.4, 22.6]	27.0 [26.4, 27.5]	13.1 [12.7, 13.5]	16.5 [16.1, 16.9]	13.99 [13.7, 14.3]	17.8 [17.35, 18.18]	13.95 [13.4, 14.5]	17.4 [16.75, 18.04]	14.3 [14.05, 14.50]	17.9 [17.64, 18.19]
**Catastrophic expenditure at 25% of non-food expenditure**												
Inpatient	0.55 [0.45, 0.66]	1.81 [1.62, 2.00]	4.10 [3.87, 4.32]	8.40 [8.06, 8.74]	1.54 [1.41, 1.68]	3.45 [3.24, 3.65]	2.11 [1.99, 2.24]	4.43 [4.20, 4.65]	1.60 [1.40, 1.81]	3.72 [3.39, 4.04]	1.98 [1.89, 2.07]	4.13 [3.99, 4.27]
Outpatient	7.26 [6., 7.6]	4.65 [4.3, 4.95]	12.0 [11.6, 12.4]	9.67 [9.3, 10.0]	9.68 [9.35, 10.00]	6.76 [6.48, 7.05]	8.61 [8.36, 8.85]	6.20 [5.93, 6.46]	10.46 [9.97, 10.96]	7.76 [7.30, 8.21]	9.63 [9.44, 982]	7.08 [6.90, 7.26]
Total OOP	8.77 [8.39, 9.16]	8.68 [8.28, 9.09]	18.1 [17.7, 18.5]	21.1 [20.6, 21.6]	12.7 [12.29, 13.02]	12.8 [12.45, 13.22]	12.5[12.20, 12.78]	13.6 [13.21, 13.95]	13.8 [13.26, 14.38]	14.3 [13.67, 14.87]	13.2 [12.95, 13.38]	13.9 [13.67, 14.16]

Source and note: same as Table 1.

OOP spending per household member (at 1999–2000 prices) was also higher in 2012 compared to 2000. Among rural households, monthly OOP spending per member was almost 60 per cent higher in 2012 compared to 2000 (INR 29.59 in 2000 versus INR 47.05 in 2012) and approximately 68 per cent higher among urban households (INR 43.28 in 2000 versus INR 72.36 in 2012) over the same period. While household OOP expenses (per member) rose both for inpatient and outpatient care, inpatient OOP spending saw faster increases than outpatient OOP spending from 2000 to 2012. These basic conclusions about trends in OOP spending do not change even when we consider OOP shares in total household expenditure. Overall, the share of OOP healthcare expenses in total household spending increased from 5.8 per cent in 2000 to 6.7 per cent in 2012.

Trends in the share of population incurring catastrophic spending were similar to those for the OOP indicators discussed above. The share of households reporting OOP spending in excess of 10% of their total expenditure increased from 15% in 2000 to 18.9% in 2012 among rural households and from 12.4% in 2000 to 15.9% in 2012 among urban households.

In Table 2, we report descriptive statistics for indicators of financial burden for the three socioeconomic categorizations considered in this paper: quintiles based on household expenditure per capita, caste and religion.

Socioeconomically disadvantaged groups, specifically the poorest 20% of households and SC/ST households, had lower OOP expenses on healthcare compared to their better-off counterparts (the richest 20% of households and ‘other caste’ households respectively). This holds for a broad range of indicators, including the share of households reporting any OOP expenditure, OOP spending per household member, the share of OOP in total spending, and measures of catastrophic spending.

### Assessing Trends: Regression results

The crude descriptive statistics in Tables 1 and 2 and associated trends are likely to be confounded by place of residence (e.g., greater proportions of Muslims (46%) live in urban areas than Hindus (37%)), region-specific differences in healthcare coverage (e.g., coverage of publicly-funded insurance programmes is higher in Southern Indian states that may be correlated with specific populations of socioeconomically disadvantaged), household age-composition and differential growth of the publicly financed RSBY program across Indian states. Thus a multivariate analysis based on equations (1) and (2) is more appropriate for assessing relative performance in the financial burden of OOP across groups over time. Tables 3 through 6 report the results from this analysis (one table for each outcome indicator), using regression results for equations (1) and (2). Although the direction of conclusions is not too different across the different specifications, our discussion of the results is based on equation (2) that controls for multiple indicators of socioeconomic status and other confounders. Throughout, estimates of other covariates are omitted from the Tables in order to economize on space, and data for the year 2000 reported in Table 2 is used as a baseline for assessing the magnitudes of changes derived from our regression analyses.

**Table 3:** Changes in households reporting any OOP and OOP as a share (%) of total household expenditure.

	Whether incurred any OOP				OOP as % of household expenditure			
	(1)			(2)	(1)			(2)
t1 (dummy for year 2005)	−3.541***	−4.376***	−5.643***	−4.586***	0.4025	0.2624	0.0896	0.2363
	[0.4279]	[0.3301]	[0.2105]	[0.4751]	[0.5199]	[0.5205]	[0.3768]	[0.6091]
t2 (dummy for year 2012)	7.135***	10.537***	10.98***	8.324***	1.332***	1.156***	1.082***	1.4561***
	[0.4099]	[0.3211]	[0.2018]	[0.4598]	[0.0692]	[0.0663]	[0.0492]	[0.0799]
quintile1 (dummy for poorest 20%)	−15.92***			−26.24***	−3.974***			−6.608***
	[0.4374]			[0.4693]	[0.1463]			[0.1504]
t1_ quintile1 (interaction term: year 2005 and poorest 20%)	−3.213***			−2.056***	−0.666			−0.8038
	[0.6047]			[0.6115]	[1.2837]			[1.2723]
t2_ quintile1 (interaction term: year 2012 and poorest 20%)	8.845***			8.43***	−0.4199**			−0.722***
	[0.5792]			[0.5865]	[0.1654]			[0.1641]
SCST (dummy for SC/ST)		−5.502***		1.598***		−0.2058**		0.3004**
		[0.3487]		[0.3694]		[0.1047]		[0.1067]
t1_ SCST (interaction term: year 2005 and SC/ST)		−2.172***		−0.917*		−0.1997		0.4101
		[0.4895]		[0.5086]		[0.9164]		[0.9214]
t2_ SCST (interaction term: year 2012 and SC/ST)		0.878**		−1.198**		0.1904*		0.5798***
		[0.4733]		[0.4874]		[0.1184]		[0.1185]
muslim (dummy for religion Muslim)			4.9295***	1.702***			−0.1165	−0.1425
			[0.4481]	[0.4534]			[0.1328]	[0.1289]
t1_ muslim (interaction term: year 2005 and Muslim)			1.7002**	2.3524***			0.4528	0.7336
			[0.6154]	[0.6155]			[1.1492]	[1.1085]
t2_ muslim (interaction term: year 2012 and Muslim)			−0.0734	0.3243			0.453***	0.9049***
			[0.5846]	[0.5820]			[0.1493]	[0.1436]
Constant	75.869***	72.155***	68.895***	74.548***	7.185***	5.433***	5.603***	2.432***
	[0.3097]	[0.2298]	[0.1523]	[0.8298]	[0.0621]	[0.0581]	[0.0439]	[0.1600]
Observations	346603	346329	346473	346299	346603	346329	346473	346299
R-squared	0.04	0.03	0.03	0.11	0.02	0.01	0.01	0.12

Notes: 1. *** significant at 1%, ** significant at 5% and * significant at 10%; 2. Standard errors in brackets; 3. Columns under (1) represent results based on equation (1) for the three socio-economic groups separately and columns under (2) represent results from equation (2) i.e. by considering all the three socio-economic groups together and other control variables; 4. The control variables used in specification (2) include household size, proportion of persons with different educational achievements, main source of livelihood of household, the ratio of males to females in the household, the proportion of children in the household, the proportion of elderly in the household, the proportion of married members in the household, and rural/urban location.

**Table 4:** Changes in households reporting inpatient OOP and inpatient OOP as a share (%) of total household expenditure.

	Whether incurred any inpatient OOP				Inpatient OOP as % of household expenditure			
	(1)			(2)	(1)			(2)
t1 (dummy for year 2005)	−8.36***	−9.75***	−9.96***	−9.789***	0.4919	0.1915	0.1168	0.4302
	[0.3324]	[0.2567]	[0.1637]	[0.3772]	[0.3501]	[0.3502]	[0.2535]	[0.4255]
t2 (dummy for year 2012)	−2.62***	−4.95***	−4.52***	−2.91***	1.363***	0.893***	0.8063***	1.4675***
	[0.3184]	[0.2497]	[0.1569]	[0.3651]	[0.0466]	[0.0446]	[0.0331]	[0.0558]
quintile1 (dummy for poorest 20%)	−10.07***			−15.04***	−1.546***			−2.611***
	[0.3397]			[0.3726]	[0.0985]			[0.1050]
t1_ quintile1 (interaction term: year 2005 and poorest 20%)	−1.813***			−1.497***	−0.6597			−0.8123
	[0.4697]			[0.4855]	[0.8645]			[0.8888]
t2_ quintile1 (interaction term: year 2012 and poorest 20%)	−2.589***			−2.137***	−1.174***			−1.299***
	[0.4499]			[0.4656]	[0.1114]			[0.1147]
SCST (dummy for SC/ST)		−2.418***		1.0261***		−0.328***		−0.0026
		[0.2712]		[0.2933]		[0.0704]		[0.0745]
t1_ SCST (interaction term: year 2005 and SC/ST)		−1.0296**		−0.2697		−0.1809		0.1746
		[0.3807]		[0.4038]		[0.6167]		[0.6437]
t2_ SCST (interaction term: year 2012 and SC/ST)		−0.611*		−0.4142		−0.1308*		0.2574**
		[0.3681]		[0.3870]		[0.0797]		[0.0828]
muslim (dummy for religion Muslim)			−0.0009	−0.9518*			−0.1594*	−0.274**
			[0.3485]	[0.3600]			[0.0894]	[0.0900]
t1_ muslim (interaction term: year 2005 and Muslim)			2.006***	2.5081***			0.3384	0.4923
			[0.4786]	[0.4887]			[0.7733]	[0.7744]
t2_ muslim (interaction term: year 2012 and Muslim)			0.5859	1.446**			−0.015	0.3099**
			[0.4546]	[0.4621]			[0.1004]	[0.1003]
Constant	24.637***	20.6011	18.59***	19.726***	1.995***	1.392***	1.293***	0.88***
	[0.2406]	[0.1787]	[0.1185]	[0.6588]	[0.0418]	[0.0391]	[0.0295]	[0.1118]
Observations	346603	346329	346473	346299	346603	346329	346473	346299
R-squared	0.03	0.01	0.01	0.06	0.02	0.01	0.02	0.05

Notes: same as Table 3.

**Table 5:** Changes in households reporting outpatient OOP and outpatient OOP as a share (%) of total household expenditure.

	Whether incurred any outpatient OOP				Outpatient OOP as % of household expenditure			
	(1)			(2)	(1)			(2)
t1 (dummy for year 2005)	0.641	0.9496**	−0.1286	0.2231	−0.0894	0.0709	−0.0272	−0.1939
	[0.4446]	[0.3427]	[0.2186]	[0.4935]	[0.3698]	[0.3671]	[0.2659]	[0.4325]
t2 (dummy for year 2012)	11.37***	16.08***	16.34***	13.043***	−0.0307	0.264***	0.276***	−0.0114
	[0.4259]	[0.3333]	[0.2095]	[0.4777]	[0.0492]	[0.0468]	[0.0347]	[0.0567]
quintile1 (dummy for poorest 20%)	−16.89***			−26.87***	−2.428***			−3.997***
	[0.4544]			[0.4875]	[0.1041]			[0.1068]
t1_ quintile1 (interaction term: year 2005 and poorest 20%)	−0.7291			0.5029	−0.0067			0.0085
	[0.6282]			[0.6352]	[0.9131]			[0.9034]
t2_ quintile1 (interaction term: year 2012 and poorest 20%)	10.598***			9.9178***	0.754***			0.5773***
	[0.6018]			[0.6093]	[0.1177]			[0.1165]
SCST (dummy for SC/ST)		−6.28***		1.0721**		0.122*		0.303***
		[0.3620]		[0.3838]		[0.0738]		[0.0758]
t1_ SCST (interaction term: year 2005 and SC/ST)		−1.539***		−0.9714*		−0.0188		0.2356
		[0.5081]		[0.5283]		[0.6464]		[0.6543]
t2_ SCST (interaction term: year 2012 and SC/ST)		1.114**		−1.1796**		0.3212***		0.3224***
		[0.4913]		[0.5063]		[0.0835]		[0.0841]
muslim (dummy for religion Muslim)			5.604***	1.9568***			0.0429	0.1314
			[0.4653]	[0.4710]			[0.0937]	[0.0915]
t1_ muslim (interaction term: year 2005 and Muslim)			0.836	1.3058**			0.1144	0.2413
			[0.6390]	[0.6394]			[0.8110]	[0.7872]
t2_ muslim (interaction term: year 2012 and Muslim)			−0.0682	0.2014			0.4681***	0.595***
			[0.6069]	[0.6046]			[0.1053]	[0.1020]
Constant	68.215***	64.231***	60.8841	67.689***	5.19***	4.041***	4.3097***	1.554***
	[0.3218]	[0.2386]	[0.1581]	[0.8620]	[0.0442]	[0.0410]	[0.0310]	[0.1136]
Observations	346603	346329	346473	346299	346603	346329	346473	346299
R-squared	0.04	0.03	0.03		0.01	0.02	0.01	0.1

Notes: same as Table 3.

**Table 6:** Percentage of household reporting catastrophic expenditure at 10 per cent of total and 25 per cent of non-food expenditure.

	Whether incurred OOP more than 10% of household expenditure				Whether incurred OOP more than 25% of non-food expenditure			
	(1)			(2)	(1)			(2)
t1 (dummy for year 2005)	1.812***	0.9879***	0.1312	1.565***	2.013***	0.9767***	0.587***	1.6489***
	[0.3503]	[0.2718]	[0.1733]	[0.3945]	[0.3326]	[0.2569]	[0.1638]	[0.3749]
t2 (dummy for year 2012)	5.107***	3.7757***	3.6453***	5.807***	2.9884***	1.0916***	0.7518***	4.1842***
	[0.3356]	[0.2643]	[0.1661]	[0.3819]	[0.3186]	[0.2498]	[0.1570]	[0.3629]
quintile1 (dummy for poorest 20%)	−13.69***			−20.96***	−9.3115***			−16.67***
	[0.3580]			[0.3897]	[0.3399]			[0.3703]
t1_ quintile1 (interaction term: year 2005 and poorest 20%)	−2.573***			−2.206***	−2.045***			−1.866***
	[0.4950]			[0.5078]	[0.4699]			[0.4826]
t2_ quintile1 (interaction term: year 2012 and poorest 20%)	−2.468***			−2.27***	−3.0786***			−2.913***
	[0.4742]			[0.4871]	[0.4502]			[0.4628]
SCST (dummy for SC/ST)		−0.897***		1.4036***		0.1706		1.0925***
		[0.2871]		[0.3068]		[0.2713]		[0.2915]
t1_ SCST (interaction term: year 2005 and SC/ST)		−1.319***		0.1163		−0.7831		0.4773
		[0.4030]		[0.4224]		[0.3809]		[0.4013]
t2_ SCST (interaction term: year 2012 and SC/ST)		−0.381***		0.1799		−0.9164*		−0.2689
		[0.3896]		[0.4048]		[0.3683]		[0.3847]
muslim (dummy for religion Muslim)			−0.3225	−0.0795			0.6569*	0.5215
			[0.3689]	[0.3765]			[0.3487]	[0.3578]
t1_ muslim (interaction term: year 2005 and Muslim)			1.0712**	1.7444***			0.9137**	1.5988***
			[0.5067]	[0.5112]			[0.4788]	[0.4857]
t2_ muslim (interaction term: year 2012 and Muslim)			−0.2035	0.7593			−0.3009	0.3729
			[0.4813]	[0.4833]			[0.4548]	[0.4593]
Constant	21.878***	13.9896***	14.275***	5.042***	18.086***	12.487***	13.163***	2.429***
	[0.2535]	[0.1892]	[0.1254]	[0.6891]	[0.2407]	[0.1788]	[0.1185]	[0.6548]
Observations	346603	346329	346473	346299	346603	346329	346473	346299
R-squared	0.02	0.01	0.01	0.07	0.01	0.01	0.01	0.06

Notes: same as Table 3.

Table 3 indicates (using our preferred set of results based on specification (2)), the poorest 20% of the households registered a faster increase in the indicator “reporting any OOP payment” compared to the richest 20% during 2000–2012. Specifically, the rise in the share of the poorest 20% reporting any OOP over this period exceeded the change in the share reporting any OOP among the richest quintile by 8.4% points (relative to a baseline share of 60.0% in 2000). Over the same period, the proportion reporting “any OOP” grew more slowly for SC/ST household (relative to non-SC/ST household) (a difference of −1.2% points, relative to a baseline of 66.7% in 2000) and the change in the proportion reporting “any OOP” remained similar for Muslim and non-Muslim households. In contrast, the share of OOP to total spending grew at a slower rate for the poorest 20% households (i.e., by −0.7% points relative to the richest 20% from a low base share of 3.2% in 2000, and faster for the SC/ST households relative to non-SC/ST households by 0.6% points (baseline share of 5.2% in 2000). Muslim households also saw their household shares of OOP to total spending increase by 0.9% points relative to non-Muslim households during 2000–12 (baseline share of 5.5% in 2000).

The results in Table 3 also allow us to look at changes over the sub-period 2000–2005. Here we observe that the change in the proportion of households reporting ‘any OOP’ was lower by 6.6% for the poorest 20% relative to the top 20% of households during 2000–2005. When taken as a share of total household spending, the poorest 20% reported slower (but statistically insignificant) growth in OOP (by −0.8 percent points) relative to the richest 20% over the same period.

SC/ST households also saw slower growth in households reporting “any OOP” (by 0.9% points) and a higher growth in the share of OOP (by 0.4% points) relative to non-SC/ST households during 2000–05, but both estimates are statistically indistinguishable from zero at the 5% level of significance. Finally, Muslim households experienced faster rates of increases in “any OOP” spending during 2000–2005 (of 2.4% points) and in the OOP share of total health spending (0.7% points, but statistically indistinguishable from zero) relative to non-Muslim households.

### Inpatient and outpatient OOP Spending

Tables 4 and 5 report results separately for OOP expenditure on inpatient and outpatient care. During 2000 to 2012, the share of households reporting ‘any OOP for inpatient care’ declined faster for the poorest 20% of households (−5.1% points, relative to a baseline of 14.6% in 2000) compared to the richest quintile (−2.9% points); the slower growth of households reporting “any OOP” in the poorest quintile also held for the sub-period 2000–2005. Moreover, the poorest 20% of households saw significantly slower increase (by −1.3% points) in the share of inpatient OOP expenses in total household spending during 2000–2012, and by 0.8% points during 2000–2005 (compared to a baseline share of 0.5% in 2000).

The findings for SC/ST households differ from those for the poorest quintile. SC/ST households also saw slower growth in the proportion reporting “any inpatient OOP” relative to non-SC/ST households in 2000–2005 and 2005–2012, but the differences (−0.3% and −0.4%, respectively) were small in magnitude and statistically insignificant. However, the share of OOP for inpatient care in total household spending for SC/ST households rose faster than non-SC/ST households in 2000–2005 and 2000–2012, by 0.2% points and 0.3% points respectively (relative to base shares of 1.1% and 1.4% for the 2 groups in 2000). Finally, Muslim households saw faster increases than non-Muslim households in both periods and for both indicators, namely “any inpatient OOP” and the share of inpatient OOP in total spending (by 1.4% points and 0.3%, respectively, relative to baseline (2000) shares of 18.6% and 1.1%, respectively).

The results on trends in Table 5 for OOP spending for outpatient care are somewhat different from those for inpatient care. The share reporting ‘any OOP spending for outpatient care’ increased faster for the poorest 20% households than for the richest 20%, by 9.9% points over the period 2000–2012. The differences between SC/ST households and non-SC/ST households in the growth of “any OOP spending on outpatient care” are rather small and also negative, −1.0% points and −1.2% points for 2000–2005 and 2005–2012, respectively (compared to base levels of 58% and 64%, respectively). A similar conclusion holds for differences in the outcome indicator “any OOP for outpatient care” for Muslim and non-Muslim households. There was no such ambiguity when the outcome was the share of OOP for outpatient care in total spending, which rose faster for the poorest 20%, SC/ST and Muslim households related to their more advantaged counterparts (richest 20%, non-SC/ST and non-Muslim) over the period 2000–2012. Moreover, the difference observed in the growth between disadvantaged and advantaged groups during 2000–2012 ranged from 0.3% to 0.6% points, which was large given the baseline shares in 2000, which ranged from 2.8% to 5.2% across all groups.

### Catastrophic expenditure

Over the period 2000 to 2012, the share of households reporting catastrophic payments increased by roughly 3% when a 10% threshold ratio of OOP spending to total household spending was used, and by roughly 1% when a threshold ratio of 25% for OOP spending to non-food spending was used (see Table 1). However, Table 6 shows that there are differences in trends across socioeconomic groups in the proportion of households incurring catastrophic spending.

Specifically, the poorest household quintile experienced a slower increase (ranging between −2.3% and 2.9% points depending on the thresholds of catastrophic payment used) in the proportion reporting catastrophic OOP compared to the richest quintile, during the period 2000–2012. However, results for SC/ST households versus non-SC/ST households show no differences in rates of change in the population share incurring catastrophic spending during 2000–2012. Finally, over the period 2000–2012, the growth in the share of Muslim households reporting catastrophic spending exceeded that of non-Muslims by 0.8% points using the 10% threshold, and by 0.4% points using the 25% catastrophic threshold, although the results did not attain statistical significance. This was in contrast to the period 2000–2005, where Muslim households experienced increases in the share reporting catastrophic spending that exceeded the increases for non-Muslim households by 1.7% points for the 10% threshold and by 1.6% points for the 25% threshold.

To assess the sensitivity of our findings to other thresholds for catastrophic spending, we undertook additional analyses using equations (1) and (2) with alternative catastrophic spending outcomes for thresholds varying between 5 per cent and 25 per cent, and considering both types of denominators: non-food spending and total spending (see Table 2 in Table S1). The results broadly confirm our findings reported in Table 6.

## Discussion and Conclusions

Our findings on trends suggest that the relative performance of the three groups of disadvantaged households that we considered (compared to their respective advantaged counterparts) in terms of the financial burden from OOP spending varies, depending on the group under consideration. The key to understanding these differences is to interpret, as noted in the methods section, a report of -“any OOP spending”- as an indicator of utilization of healthcare services, Multiple studies have relied on this interpretation of the indicator variable for OOP spending and evidence from household surveys in India containing information both on healthcare use and OOP shows that the indicator variables for reporting any OOP spending and any utilization are highly correlated, with the correlation coefficient exceeding 0.95, both for inpatient care and outpatient care. This is not surprising because even when care is heavily subsidized (or free), drugs have to be purchased by households.

Consider first the position of the poorest 20% of households, relative to the better-off households. Overall, there was an increase in households reporting “any OOP” during 2000–2012, but it is more insightful to examine whether a household reported “any OOP for outpatient care” or “any OOP for inpatient care”. We find that while the rise in the share of households reporting “any OOP for outpatient care” among the poorest 20% households exceeded that for the richest 20% substantially (by 8.4% points, given a baseline shares of 51.3% and 68.2% respectively in 2000), the opposite was true for “any OOP for inpatient care”. Our interpretation is that the poorest 20% of households saw increased utilization of outpatient care and lower utilization for inpatient care, relative to the top 20% of households. This finding is consistent with some earlier research in India suggesting lower access to inpatient care among the poor even after the introduction of publicly financed insurance plans for inpatient care specifically targeting them, because households may have to buy medicines from the outside for an inpatient at a hospital [28]. However, it is possible that a large part of increased use of inpatient care by poorer groups may end up not being captured by expenditure data (CES) because of the availability of the “cashless” schemes such as RSBY and *Arogyasri* in recent years, exclusively for inpatient care.

The slower relative rise in the share of overall (combined inpatient and outpatient) OOP spending in total household spending as well in the share of OOP inpatient spending (in total household spending) for the poorest 20% over the period 2000–2012 is potentially also consistent with either of the two interpretations noted in the preceding paragraph. For instance, a rising share of OOP spending for outpatient care in total household spending for the poorest 20% (relative to the richest 20%) can be expected when inpatient insurance cover increases, allowing households to shift more of their resources to necessary outpatient care, while increasing the use of (cheaper) inpatient care. It is also possible that rising costs (relative to the top 20%) of inpatient care coupled with inadequate insurance coverage lead poor households to substitute away from inpatient care and use more outpatient care. However, given that overall OOP as a share of household expenditure rose during this period (Table 1), and our measure for inpatient utilization actually fell for the bottom 20% (Table 1), the case for a relative decline in access to inpatient care due to its becoming more expensive appears strong.

For SC/ST households, the conclusions suggest little or no-change in their relative position with respect to utilization, but an increased financial burden, relative to non-SC/ST households in the form of OOP shares of total household spending. Although the proportion of SC/ST households reporting any OOP, any inpatient OOP and any outpatient OOP rose less quickly than for non-SC/ST households, the differences were often statistically insignificant and their magnitude small (−1.2% during 2000–2012 relative to a base of 58.0% for reporting any outpatient OOP in 2000; and −0.4% during 2000–2012 relative to a base of 18.2% for reporting any inpatient OOP in 2000). Relative to non-SC/ST households, their utilization of healthcare services is therefore, unlikely to have improved. Our analyses also show, however, that OOP rose as a share of total household spending faster for SC/ST households relative to non-SC/STs households, irrespective of whether we consider overall OOP, OOP for inpatient care, or OOP for outpatient care (the coefficients for indicators of catastrophic spending were also mostly positive, but all statistically indistinguishable from zero). These relative increases in the share of OOP spending in total SC/ST household spending were non-trivial.

Muslim households experienced small and statistically insignificant increases in the proportion reporting any OOP for overall spending and for outpatient spending, relative to non-Muslim households. However, the share of Muslim households reporting any inpatient spending declined by 1.4% points less than for non-Muslim households. At the same time the share of OOP in total household spending increased sharply among Muslims (relative to non-Muslims) in all three of the indicators of OOP shares – outpatient care, inpatient care and overall – so their OOP spending burden also increased. Because a slew of policy interventions were introduced in the period after 2005, it is of interest whether there were differences in (relative) outcomes for the sub-period 2005–12. The difference in the coefficients for the interaction terms in the 2 periods in specification (2) allows us to obtain information on trends in 2005–12. It can be readily checked that our central findings are broadly the same as for the period 2000–2012. While we are unaware of any previous analyses that take into account the relative position of Muslim households with respect to the financial burden, our findings for SC/ST households are similar to those reported in Fan et al. [29] in their work on the *Arogyasri* scheme in Andhra Pradesh.

Overall, our analysis highlights a number of areas of concern. For the poorest 20% and SC/ST households, the lack of evidence for increased utilization of inpatient care (relative to the top 20% of households) is puzzling in light of the rapid expansion of publicly financed insurance schemes for hospital-based services, such as RSBY and *Arogyasri*. Among SC/ST and Muslim households that experienced roughly similar changes in most indicators of utilization, it is concerning that they experienced sharper increases in OOP expenses compared to their better-off counterparts. These trends and the evidence for rising shares of OOP spending for outpatient care among all three groups are of spme interest given large government investments in NRHM during this period. To recall from earlier in the paper, NRHM involved a large scale provision and financing intervention intended to make healthcare available in rural India through public sector provision, mostly in outpatient settings.

Some recent studies for India have found that health care utilization increased significantly following the introduction of the NRHM and RSBY programs and other state-level health insurance initiatives [30], [31]. However, this is not what we see from the data on relative change in indicators for “any healthcare OOP.” What we see instead is a rising share of OOP in total household spending among disadvantaged households, relative to their better-off counterparts. We suspect these findings reflect serious gaps in existing programs with regard to access to affordable outpatient care and drugs which account for the bulk of OOP spending on healthcare in India (Table 1). For example, few state-level governments in India have been able to provide access to subsidized drugs, so households are likely to have been forced to pay for medicines from their own pocket. As drug prices in the open market have increased substantially over the years in India, the overall OOP burden is also likely to have increased [32].

Apart from budgetary limitations, some of the government programs have ended up with a narrower focus than their original mandate. For instance, the NRHM was primarily intended to strengthen existing public health facilities and to facilitate the provision of free or highly subsidised health care services. However, it has effectively ended up focusing on reproductive and child health (RCH) interventions. The relative neglect of other elements of primary care, including management of chronic conditions, is likely to have translated into increased financial burden on poor and other less advantaged population groups who needed access to other types of care, especially range of primary healthcare [33], [34].

Publicly financed insurance coverage, despite its rapid expansion in population coverage, is relatively limited in the financial benefits it offers. For instance, the benefits package under the largest of these schemes (RSBY) has been very modest (only up to a maximum of INR 30,000 per family, equivalent to approximately US $ 500 per annum) and limited up to five members in a family). These schemes may facilitate healthcare use, but they may also facilitate additional (OOP) contributions due to the limited financial cover, especially if the demand for health service use exceeds the approved upper limit for coverage and also because coverage of post-hospitalization care is limited. If the cost of complementary items such as drugs were to increase sharply, inpatient care use may also fall, as seems to have occurred in the case of the poorest 20% of the households. Supplier induced utilisation over and above the approved limit may be a factor here, although it is very difficult to test this hypothesis.

Healthcare provision and financing innovations are possibly poorly targeted. Earlier research for India has shown that in the absence of effective targeting, benefits from public services will disproportionately flow to richer groups [9], [35]. One potential driver of this outcome is the reliance of existing schemes on the so-called “BPL (Below Poverty Line) lists” prepared by different state governments based on survey data in the year 2002–03 to identify program beneficiaries. It is well known that this BPL list suffers from a high degree of ‘exclusion’ and ‘inclusion errors’ with obvious implications for our findings [36]–[38]. Existing literature also suggests that SC/ST populations tend to benefit less from public programs than other population sub-groups [35] consistent with our findings, pointing to systematic challenges in reaching this group. We could not locate equivalent evidence for Muslims’ access to public healthcare services in earlier literature, but recent work [23] as well as lack of specific emphasis on reaching Muslim households under RSBY and NRHM could explain the findings on the relative performance of Muslim households.

Our study has obvious limitations. Focusing solely on OOP payments for healthcare has its weaknesses in assessing the financial burden of illness. Illness can also translate into income losses and sale of productive assets [3], [9] and these do not readily show up in OOP expense data. Our approach to catastrophic headcount used in this paper has also been a subject of considerable recent debate among researchers and although consistent with the vast majority of the literature [39], [40] is somewhat simplistic, and lacking a theoretical explanation We tried to address this concern by using different thresholds of OOP as a share of total and non-food expenditure, finding that the use of different thresholds does not affect our main conclusions. Another source of concern is that our analysis is based on health expense questions in household consumer expenditure survey data and these often tend to underestimate health spending. This need not be a problem, however, if underreporting is reasonably stable across time and sub-groups. A final concern relates to differences in health status of population across SES groups that can confound our conclusions on trends in OOP spending. Our data, from consumer expenditure surveys, did not include any information on health status. However, we partially addressed this concern by controlling separately for the shares of young children (age 5 years or less) and elderly (age 60 years and over) in the household in our regression. It is well known that healthcare use is high in both these age groups and indeed our analysis showed that households with children (age 5 years or less) and elderly (age 60 years and over) had higher growth in OOP as a share of household expenditure, relative to households with no young children and elderly members.

## Supporting Information

Table S1 (DOCX)
