# Supplementary material for: Moving to Universal Coverage? Trends in the Burden of Out-Of-Pocket Payments for Health Care across Social Groups in India, 1999–2000 to 2011–12
Source: PLoS One. 2014 Aug 15;9(8):e105162. doi: 10.1371/journal.pone.0105162 (PMC4134256; doi:10.1371/journal.pone.0105162)
Supplement: Table S1 — (DOCX) [file pone.0105162.s001.docx]

**Table S1: Mean and standard deviations of households with different socio-economic characteristics**

| Variable | 1999-2000 | | 2004-05 | | 2011-12 | |
| --- | --- | --- | --- | --- | --- | --- |
|  | Mean | SD | Mean | SD | Mean | SD |
| Quintile1 | 0.2006 | 0.4005 | 0.2000 | 0.4000 | 0.2000 | 0.4000 |
| Quintile2 | 0.2011 | 0.4008 | 0.2000 | 0.4000 | 0.2000 | 0.4000 |
| Quintile3 | 0.1986 | 0.3989 | 0.2000 | 0.4000 | 0.2000 | 0.4000 |
| Quintile4 | 0.2002 | 0.4002 | 0.2000 | 0.4000 | 0.2000 | 0.4000 |
| Quintile5 | 0.1995 | 0.3996 | 0.2000 | 0.4000 | 0.2000 | 0.4000 |
|  |  |  |  |  |  |  |
| caste1 (ST) | 0.0888 | 0.2845 | 0.0877 | 0.2829 | 0.0892 | 0.2851 |
| caste2 (SC) | 0.1925 | 0.3943 | 0.1970 | 0.3978 | 0.1902 | 0.3925 |
| caste3 (OBC) | 0.3522 | 0.4777 | 0.4015 | 0.4902 | 0.4308 | 0.4952 |
| caste4 (Other) | 0.3664 | 0.4818 | 0.3137 | 0.4640 | 0.2898 | 0.4537 |
|  |  |  |  |  |  |  |
| edu1 (Illiterate) | 0.3168 | 0.4652 | 0.2253 | 0.4178 | 0.1730 | 0.3782 |
| edu2 (Primary) | 0.1428 | 0.3499 | 0.1610 | 0.3675 | 0.1174 | 0.3219 |
| edu3 (Middle) | 0.1921 | 0.3940 | 0.2274 | 0.4192 | 0.1945 | 0.3958 |
| edu4 (Higher secondary) | 0.2408 | 0.4276 | 0.2666 | 0.4422 | 0.3534 | 0.4780 |
| edu5 (Graduate and above) | 0.1074 | 0.3097 | 0.1196 | 0.3245 | 0.1617 | 0.3682 |
|  |  |  |  |  |  |  |
| religion1 (Hindu) | 0.8335 | 0.3725 | 0.8338 | 0.3722 | 0.8317 | 0.3741 |
| religion2 (Muslim) | 0.1089 | 0.3115 | 0.1124 | 0.3158 | 0.1181 | 0.3228 |
| religion 3 (Christian) | 0.0272 | 0.1626 | 0.0240 | 0.1532 | 0.0245 | 0.1547 |
| religion 4 (Other) | 0.0304 | 0.1718 | 0.0298 | 0.1699 | 0.0256 | 0.1579 |
|  |  |  |  |  |  |  |
| Household size<5 | 0.6634 | 0.4726 | 0.6913 | 0.4620 | 0.7433 | 0.4368 |
| Proportion female | 0.4761 | 0.2056 | 0.4791 | 0.2047 | 0.4767 | 0.2108 |
| Proportion married | 0.4659 | 0.2564 | 0.4751 | 0.2606 | 0.4903 | 0.2672 |
|  |  |  |  |  |  |  |
| Age 0-4 years | 0.0928 | 0.1395 | 0.0863 | 0.1379 | 0.0693 | 0.1274 |
| Age 5-14 years | 0.2130 | 0.2182 | 0.2027 | 0.2185 | 0.1816 | 0.2137 |
| Age 15-29 years | 0.2725 | 0.2539 | 0.2729 | 0.2559 | 0.2796 | 0.2657 |
| Age 30-59 years | 0.3341 | 0.2352 | 0.3474 | 0.2375 | 0.3700 | 0.2457 |
| Age 60 years and above | 0.0876 | 0.1981 | 0.0907 | 0.2051 | 0.0994 | 0.2181 |
|  |  |  |  |  |  |  |
| Self-employed in non-agriculture | 0.1898 | 0.3921 | 0.2166 | 0.4119 | 0.2180 | 0.4129 |
| Self-employed in agriculture | 0.2431 | 0.4289 | 0.2574 | 0.4372 | 0.2366 | 0.4250 |
| Regular wage earners | 0.1126 | 0.3161 | 0.1123 | 0.3158 | 0.1911 | 0.3932 |
| Casual wage earners | 0.3374 | 0.4728 | 0.3033 | 0.4597 | 0.2766 | 0.4473 |
| Others | 0.1172 | 0.3217 | 0.1104 | 0.3134 | 0.0777 | 0.2677 |
|  |  |  |  |  |  |  |
| Rural | 0.7279 | 0.4450 | 0.7250 | 0.4465 | 0.6875 | 0.4635 |
| Number of observations | 120297 | | 124644 | | 101662 | |

Source same as Table 1.

**Table S2: Percentage of household reporting OOP share being above different thresholds of total and non-food expenditure.**

|  | thresholds (%) of household expenditure | | | | | thresholds (%) of non-food expenditure | | | | |
| --- | --- | --- | --- | --- | --- | --- | --- | --- | --- | --- |
|  | 5% | 10% | 15% | 20% | 25% | 5% | 10% | 15% | 20% | 25% |
| t1 (dummy for year 2005) | -1.069** | 1.565*** | 1.891*** | 2.1029*** | 1.7886*** | -3.33*** | -2.13*** | -0.1232 | 0.9662** | 1.649*** |
|  | [0.5003] | [0.3945] | [0.3118] | [0.2521] | [0.2055] | [0.5375] | [0.5177] | [0.4704] | [0.4206] | [0.3749] |
| t2 (dummy for year 2012) | 5.251*** | 5.8066*** | 5.454*** | 4.912*** | 4.0967*** | 1.4242*** | 1.8*** | 3.1799*** | 4.0166*** | 4.184*** |
|  | [0.4843] | [0.3819] | [0.3018] | [0.2440] | [0.1989] | [0.5202] | [0.5011] | [0.4553] | [0.4071] | [0.3629] |
| quintile1 (dummy for poorest 20%) | -26.28*** | -20.96*** | -16.52*** | -13.02*** | -9.989*** | -21.40*** | -21.44*** | -20.56*** | -18.81*** | -16.67*** |
|  | [0.4942] | [0.3897] | [0.3080] | [0.2491] | [0.2030] | [0.5310] | [0.5114] | [0.4646] | [0.4155] | [0.3703] |
| t1_ quintile1 (interaction term: year 2005 and poorest 20%) | -1.3903** | -2.206*** | -2.354*** | -2.319*** | -1.961*** | -1.7688** | -0.5216 | -1.1218* | -0.9866* | -1.866*** |
|  | [0.6440] | [0.5078] | [0.4014] | [0.3245] | [0.2645] | [0.6919] | [0.6664] | [0.6054] | [0.5414] | [0.4826] |
| t2_ quintile1 (interaction term: year 2012 and poorest 20%) | 3.023*** | -2.27*** | -3.610*** | -3.663*** | -3.537*** | 5.2048*** | 2.6116*** | -0.1046 | -1.963*** | -2.913*** |
|  | [0.6177] | [0.4871] | [0.3849] | [0.3113] | [0.2537] | [0.6636] | [0.6391] | [0.5807] | [0.5192] | [0.4628] |
| SCST (dummy for SC/ST) | 1.2104*** | 1.404*** | 0.9309*** | 0.6222*** | 0.6831*** | 0.9361** | 0.8557** | 1.3057*** | 1.5277*** | 1.093*** |
|  | [0.3891] | [0.3068] | [0.2425] | [0.1961] | [0.1598] | [0.4180] | [0.4026] | [0.3658] | [0.3271] | [0.2915] |
| t1_ SCST (interaction term: year 2005 and SC/ST) | 0.2377 | 0.1163 | 0.4689 | 0.5306** | 0.4368** | 0.0812 | 0.2198 | 0.0562 | -0.0881 | 0.4773 |
|  | [0.5356] | [0.4224] | [0.3338] | [0.2699] | [0.2200] | [0.5754] | [0.5542] | [0.5035] | [0.4502] | [0.4013] |
| t2_ SCST (interaction term: year 2012 and SC/ST) | 1.0204** | 0.1799 | -0.2062 | 0.2139 | 0.0443 | 1.1824** | 0.4519 | -0.3477 | -0.7078* | -0.2689 |
|  | [0.5133] | [0.4048] | [0.3199] | [0.2587] | [0.2109] | [0.5515] | [0.5311] | [0.4826] | [0.4315] | [0.3847] |
| muslim (dummy for religion Muslim) | 0.1679 | -0.0795 | -0.2152 | -0.2634 | -0.0941 | 2.5705*** | 2.7095*** | 1.6915*** | 1.6334*** | 0.5215 |
|  | [0.4775] | [0.3765] | [0.2976] | [0.2406] | [0.1961] | [0.5130] | [0.4940] | [0.4489] | [0.4014] | [0.3578] |
| t1_ muslim (interaction term: year 2005 and muslim) | 1.935*** | 1.744*** | 1.4189*** | 0.9689*** | 0.363 | 0.5709 | -0.0758 | 0.5388 | 0.6672 | 1.599*** |
|  | [0.6482] | [0.5112] | [0.4040] | [0.3267] | [0.2663] | [0.6964] | [0.6707] | [0.6094] | [0.5449] | [0.4857] |
| t2_ muslim (interaction term: year 2012 and muslim) | 0.4106 | 0.7593** | 0.2571 | 0.6757** | 0.2432 | 0.4115 | 0.0876 | -0.1974 | -0.4552 | 0.3729 |
|  | [0.6130] | [0.4833] | [0.3820] | [0.3089] | [0.2518] | [0.6585] | [0.6342] | [0.5762] | [0.5153] | [0.4593] |
| Constant | 13.645*** | 5.042*** | 2.759*** | 1.426*** | 1.0007*** | 29.58*** | 13.52*** | 5.712*** | 3.3*** | 2.429*** |
|  | [0.8739] | [0.6891] | [0.5446] | [0.4404] | [0.3589] | [0.9388] | [0.9042] | [0.8215] | [0.7346] | [0.6548] |
| Observations | 346299 | 346299 | 346299 | 346299 | 346299 | 346299 | 346299 | 346299 | 346299 | 346299 |
| R-squared | 0.08 | 0.07 | 0.06 | 0.05 | 0.05 | 0.08 | 0.07 | 0.07 | 0.06 | 0.06 |

Notes: same as notes 1,2 and 4 in Table 3
